# Bioactive Properties of *Campomanesia lineatifolia*: Correlation Between Anti-*Helicobacter pylori* Activity, Antioxidant Potential and Chemical Composition

**DOI:** 10.3390/plants13223117

**Published:** 2024-11-05

**Authors:** Nívea Cristina Vieira Neves, Morgana Pinheiro de Mello, Sinéad Marian Smith, Fabio Boylan, Marcelo Vidigal Caliari, Rachel Oliveira Castilho

**Affiliations:** 1 GnosiaH, Laboratório de Farmacognosia, Faculdade de Farmácia, Universidade Federal de Minas Gerais, Belo Horizonte 31270-901, Brazil; morganamello19@gmail.com; 2 School of Pharmacy and Pharmaceutical Sciences, Trinity Biomedical Institute, Trinity College Dublin, Dublin 2, D02 PN40 Dublin, Ireland; fabio.boylan@tcd.ie; 3 Departamento de Farmácia, Centro Universitário Santa Rita, Conselheiro Lafaiete 36408-899, Brazil; 4 Department of Clinical Medicine, School of Medicine, Trinity College Dublin, Trinity Centre, Tallaght University Hospital, Dublin 24, D24 NR0A Dublin, Ireland; 5 Departamento de Patologia Geral, Instituto de Ciências Biológicas, Universidade Federal de Minas Gerais, Belo Horizonte 31270-901, Brazil; caliari@icb.ufmg.br; 6 Consórcio Acadêmico Brasileiro de Saúde Integrativa, CABSIN, São Paulo 05449-070, Brazil

**Keywords:** Campomanesia, *Campomanesia lineatifolia*, Myrtaceae, gabiroba, *Helicobacter pylori*, antimicrobial, DPPH, antioxidant, quercitrin, myricitrin

## Abstract

*Helicobacter pylori* is found in the stomach of patients with chronic gastritis and peptic ulcers, infecting approximately half of the world’s population. Current treatment for *H. pylori* infection involves a multi-drug therapeutic regime with various adverse effects, which leads to treatment abandonment and contributes to the emergence of resistant strains of *H. pylori*. Previously, we demonstrated that the essential oil of *Campomanesia lineatifolia* leaves exhibited an anti-*H. pylori* activity. In this study, we aimed to evaluate the phenolic content of the phenolic-rich ethanol extract (PEE) from *C. lineatifolia* and its anti-*H. pylori* and antioxidant properties. Additionally, the anti-*H. pylori* activity was assessed in polar and non-polar fractions from PEE, isolated myricitrin (MYR) and a mixture of myricitrin and quercitrin (MYR/QUER) from polar fractions, and aqueous extract (tea) to correlate the responsible fractions or compounds with the observed activity. Broth microdilution assays were performed to assess the anti-*H. pylori* activity using type cultures (ATCC 49503, NCTC 11638, both clarithromycin-sensitive) and clinical isolate strains (SSR359, clarithromycin-sensitive, and SSR366, clarithromycin-resistant). The antioxidant activity was evaluated using the DPPH assay. The total tannin and flavonoid contents were determined using the hide-powder method, the Folin-Ciocalteu reagent, and the aluminium chloride colourimetric assay, respectively. The tea (MIC 1:100), PEE, polar and non-polar fractions, MYR, and MYR/QUER inhibited the growth of *H. pylori* strains tested (MIC values ranging from 0.49 to 250 μg/mL). The antioxidant assays revealed that PEE exhibited a higher antioxidant activity (EC_50_ = 18.47 μg/mL), which correlated to the high phenolic content (tannin and flavonoid, 22.31 and 0.15% *w*/*w*, respectively). These findings support the traditional use of *C. lineatifolia* as a multitarget medicinal plant for treating gastric ulcers and reinforce the potential use of the species as a coadjuvant in therapeutic regimes involving patients with resistant *H. pylori* infection.

## 1. Introduction

*Helicobacter pylori* is the most frequent cause of chronic gastritis, which can develop into peptic ulcer and gastric cancer [1]. It is estimated that *H. pylori* infects almost half the world’s population [2], with significant differences between geographical areas. In many developed countries with decreasing prevalence of *H. pylori* infection and increased curing of ulcer patients, the proportion of all peptic ulcers caused by this bacterium is falling [3]. However, in developing countries, infections by *H. pylori* are ubiquitous, reaching the highest rate of 80% or more among adults in Africa, followed by Latin America (63.4%) and Asia (54.7%), which may contribute to increased morbidity and mortality worldwide [4].

Current treatment of *H. pylori* infection involves a combination of an antisecretory drug, such as proton pump inhibitors (PPI), together with two antibiotics (triple therapy) and bismuth (bismuth quadruple therapy) [4]. Almost all World Health Organization (WHO) regions have a resistance rate of more than 15% to clarithromycin, metronidazole, and levofloxacin [5]. In this scenario, adjuvant therapies have been highlighted as successful options for treating *H. pylori* infections. Medicinal plants have shown efficacy in treating gastric ulcers with fewer side effects and lower recurrence rates [6]. Moreover, combining herbal and conventional medicines against gastric ulcers shows a synergistic effect against their treatment [7,8].

*Campomanesia lineatifolia* Ruiz and Pavón is a native Brazilian species found in the Amazon Rainforest, Atlantic Forest, Cerrado and Caatinga. It is one of several Brazilian species of Myrtaceae known by the popular name “gabiroba” [9,10,11]. In ethnopharmacological use of *C. lineatifolia*, it is reported as an antimicrobial agent [12] and antiemetic, against hemoptysis and skin infection [13,14]. Other species of the *Campomanesia* genus are used in traditional medicine as an infusion to treat gastrointestinal disorders, such as diarrhoea and stomach pains [15], and the antiulcer and gastroprotective biological activities are described in the literature for *C. reitziana* and *C. xanthocarpa*, respectively [16,17]. Considering the wide distribution of *Campomanesia* species in the Brazilian territory [10], added to their traditional use in popular medicine for the treatment of various diseases, the study of the *C. lineatifolia* species is extremely relevant, emerging as efficacious, safe, and widely available alternative therapies for peptic ulcers.

Previous results showed that *C. lineatifolia* leaf essential oil exhibits antimicrobial activity against sensitive and resistant clarithromycin *H. pylori* strains [18]. Additionally, the phenolic-rich ethanol extract from *C. lineatifolia* leaves (PEE) inhibited the NF-κB/TNF-α signalling pathway, suggesting a possible anti-inflammatory activity of PEE that could justify the ethnopharmacological use of the species [19,20].

Considering the pathogenesis of gastric ulcers due to *H. pylori* infection, we proposed a study to investigate the anti-*H. pylori* activity of the leaf extract of *C. lineatifolia*. Our study aimed to determine whether the phenolic-rich ethanol extract, fractions, isolated flavonoids, and tea have anti-*H. pylori* activity. We also aimed to correlate the phenolic content of the extract with its anti-*H. pylori* and antioxidant properties. This study investigated the anti-*H. pylori* activity of *C. lineatifolia* for the first time and holds the potential to provide valuable insights into the use of medicinal plants in the treatment of *H. pylori* infections, sparking interest and intrigue among the scientific community.

## 2. Results

### 2.1. Extraction and Chemical Profile of PEE, Fractions and Isolated Flavonols

To obtain a phenolic-rich extract, an experimental design was carried out using different solvents and mixtures of solvents, as well as different extraction methods. The extractive method by maceration under a continuous reflux system using 96° ethanol as the solvent showed the best concentration of the main phenolics and is, therefore, considered optimised (PEE) [19].

**Figure 1:**
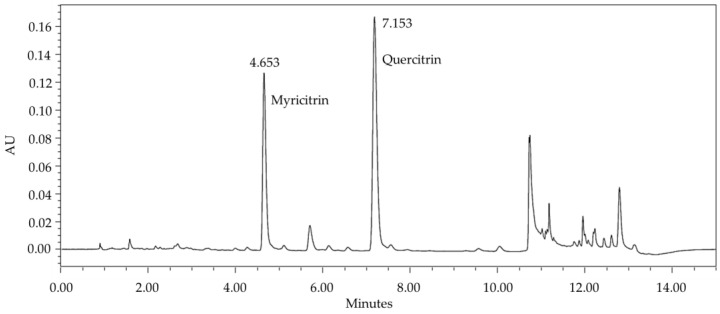
Chromatographic profile obtained by UHPLC-UV-DAD, in λ = 335 nm, of phenolic-rich ethanol extract from *Campomanesia lineatifolia* leaves. Legend: UHPLC-UV-DAD = ultra-high performance liquid chromatography coupled to ultraviolet–photodiode array detector.

The chemical profile of our Optimised Extraction Method (PEE), obtained through ultra-high-performance liquid chromatography (UHPLC), revealed a treasure trove of phenolic compounds. The extract is not just rich, but abundant in phenolics, flavonoids, and tannins. In order to develop an analytical method for the chemical and biological standardisation of PEE, quercitrin and myricitrin were selected as chemical markers, as they are the majority flavonoids, as depicted in Figure 1. In an initial scan, a significant improvement in the resolution of the chromatogram was observed when the reading was carried out at a wavelength of 335 nm, as this is more selective for flavonoids [21].

In a previous study, to identify the other chemical markers through high-speed countercurrent chromatography (HSCCC), the PEE was fractionated using hexane: ethyl acetate: methanol: water (HEMWat) in a ratio of 1:4:1:4 to obtain the upper and lower phases, or non-polar and polar fractions, respectively. This fractionation technique combined with high-performance liquid chromatography coupled to electrospray ionisation and quadrupole time-of-flight mass spectrometry (HPLC-ESI-QTOF-MS), and nuclear magnetic resonance (NMR), enabled the identification of fourteen chemical compounds. Considering that myricitrin and quercitrin were the main flavonols identified in the PEE fractions, after attempts to purify these compounds, the isolation of myricitrin (MYR) and a mixture of myricitrin plus quercitrin (MYR/QUER) was achieved [20]. These fractions and main isolated compounds were used in the present study to identify the compounds responsible for the anti-*H. pylori* activity.

Finally, to assess the anti-*H. pylori* activity, the dried plant drug was extracted by water infusion to mimic the pharmaceutical form traditionally used by the population for gastritis and ulcers, i.e., tea [15,17,20].

### 2.2. Tannin and Flavonoid Content

Given that the main compounds of *C. lineatifolia* are phenolics, the quantification of phenolic, tannin, and flavonoid contents in the PEE is of significant importance. This was achieved using spectrophotometric methods. The tannin content was estimated by calculating the difference between the total phenolic content and polyphenols not adsorbed by the hide-powder method. Pyrogallol served as a standard for constructing the analytical curve, with the equation of the standard curve obtained as follows:

A = 6.4544 C + 0.0778(1)

where A is the absorbance of pyrogallol at λ 715 nm, and C is the concentration (mg/mL), subsequently converted to % *w*/*w*. Correlation coefficient, R^2^ = 0.9976.

The concentration of total phenolic content in the PEE of *C. lineatifolia* was measured with precision at 25.410 ± 1.039, and polyphenols not adsorbed by the hide-powder were found to be 3.100 ± 0.631. The total tannin content was also determined with high accuracy at 22.310 ± 1.431. All the concentrations were expressed as grams of pyrogallol equivalents per 100 g of samples (% *w*/*w*). These values represent the mean ± SD of triplicate experiments, further ensuring the reliability of the data.

The flavonoid content was determined by using an aluminium chloride colourimetric assay using the acid hydrolysis procedure. Quercetin was used as a standard for analytical curve construction. The equation of the standard curve obtained was:

A = 0.0415 C + 0.0063(2)

where A is the absorbance of quercetin at λ 425 nm, and C is the concentration (μg/mL), subsequently converted to % *w*/*w*. Correlation coefficient, R^2^ = 0.9949.

The total of flavonoid content was 0.1536 ± 0.009, expressed as grams of quercetin equivalents per 100 g of samples (% *w*/*w*).

### 2.3. Antioxidant Activity

**Figure 2:**
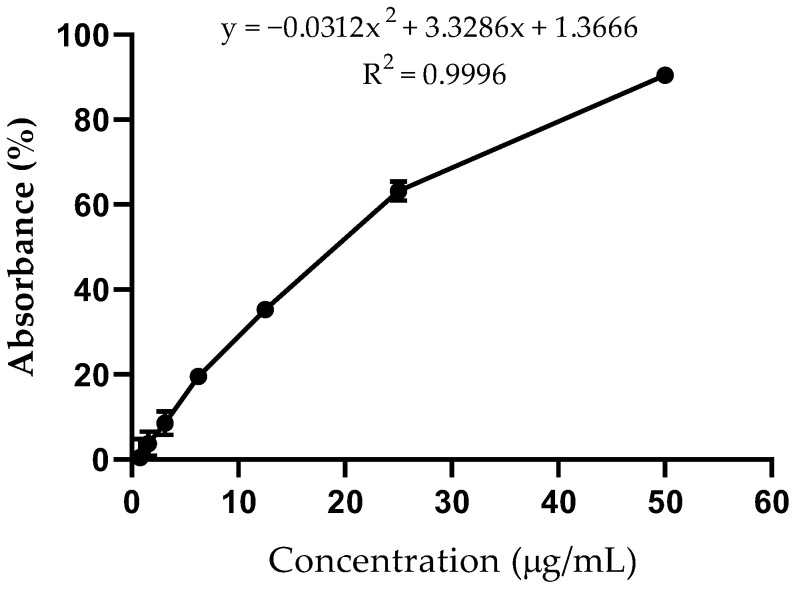
Antioxidant activity of phenolic-rich ethanol extract (PEE) of *Campomanesia lineatifolia*. Each value represents the mean ± SD of triplicate experiments.

The DPPH radical scavenging assay, a widely used method for evaluating the free radical scavenging activity of antioxidants, is known for its simplicity and high sensitivity. The results (Figure 2) indicate that the PEE exhibited a high antioxidant activity (EC_50_ = 18.47 ± 3.74 μg/mL).

### 2.4. Anti-Helicobacter pylori Activity

The antibacterial activities against multiple *H. pylori* strains of PEE, polar and non-polar fractions, isolated myricitrin, myricitrin/quercitrin mixture, and the tea were compared by determining their MICs through the broth microdilution method (Table 1).

The PEE showed an inhibitory activity for all the *H. pylori* strains tested, with MIC values ranging from 0.49 to 250 μg/mL.

The polar fraction compared to the non-polar fraction showed similar inhibition of the clarithromycin-sensitive clinical isolate (125 μg/mL). It is noted that the non-polar fraction presented better activity (125 μg/mL) for the clarithromycin-resistant clinical isolate than the polar fraction (250 μg/mL). Of the two *H. pylori* clarithromycin-sensitive type cultures, both polar and non-polar fractions were able to inhibit the growth with MIC values ranging from 0.49 to 500 μg/mL.

The isolated myricitrin compared to the myricitrin+quercitrin mixture, the chemical markers, showed good activity against the two *H. pylori* clarithromycin-sensitive type cultures and the clarithromycin-resistant clinical isolate, with MICs ranging from 62.5 to 250 μg/mL. On the other hand, MYR/QUER gave a MIC value of 31.25 μg/mL for the clarithromycin-sensitive clinical isolate.

Among the *C. lineatifolia* samples tested, it can be seen that the tea was the one that showed the best inhibitory responses, considering that it was able to inhibit all the *H. pylori* strains at the lowest concentration (1:100).

## 3. Discussion

Previous studies demonstrated the ethanolic extract’s high phenolic content from *C. lineatifolia* leaves and its ethyl acetate fraction, as well as high antioxidant activity [22,23]. Briefly, the phytochemical studies revealed the presence of flavonoids and tannins. Catechin and quercitrin were identified by bioguided chromatographic fractionation of the ethyl acetate fraction. In addition, the ethanolic extract and the ethyl acetate fraction presented in vitro antioxidant activity and promoted gastroprotection in experimental in vivo models of ethanol- and indomethacin-induced gastric lesions.

Considering that in *H. pylori* infections, excessive production of reactive oxygen and nitrogen species is observed, one can infer that medicinal plants with high antioxidant activity may be effective in treatment. These free radicals correlate with the formation of damage to the gastric mucosa and also contribute to bacterial colonisation [24]. It is expected that, due to its high antioxidant power, *C. lineatifolia* extract could contribute to gastroprotection and promote the inhibition of *H. pylori* growth. Therefore, Neves et al. [19] proposed optimising the extraction of *C. lineatifolia* leaves to obtain a phenolic-rich ethanolic extract (PEE) in an extractive method of simpler, faster and lower-cost execution using green solvents, which could have high antioxidant activity. As described above, the extract and fraction of *C. lineatifolia* contain tannins and flavonoids, so we aimed to conduct these analyses in the PEE.

In this study, the total phenolic, tannin and flavonoid content was 25.410 ± 1.039%, 22.310 ± 1.431%, and 0.1536 ± 0.009%, respectively. In comparison, Madalosso et al. [22] demonstrated that the ethanol extract obtained by percolating *C. lineatifolia* leaves had the lowest tannin content, 18.7 ± 0.1%, but a higher flavonoid content, 4.0 ± 0.1%. These results indicate that the optimised extraction of *C. lineatifolia* leaves described by Neves et al. [19] was an efficient method for extracting phenolics. It is important to note that although the total phenolic and tannin content was higher, the flavonoid content was lower than these percolation extraction methods. This can be explained by the fact that the two main flavonoid markers, quercetrin and myricitrin, were used to validate the analytical method but not the total flavonoid content. Indeed, when comparing the chromatographic profiles of the ethanolic extract obtained by percolation and the optimised extract, the PEE showed significantly higher amounts of these flavonoid markers [19]. Moreover, the low concentration of total flavonoid content can be explained by various factors related to the plant’s stage of development, seasonality, temperature, water availability and other factors [25].

Phenolic compounds are recognized as one of the plants’ most important classes responsible for their antioxidant capacity [26,27]. The antioxidant activity measured by the bleaching of the DPPH radical is expressed as EC_50_ (μg/mL), which denotes the concentrations of sample required to scavenge 50% of DPPH free radicals. If EC_50_ ≤ 50 μg/mL, the sample has high antioxidant capacity; if 50 μg/mL < EC_50_ ≤ 100 μg/mL, the sample has moderate antioxidant capacity; and if EC_50_ > 200 μg/mL, the sample has no relevant antioxidant capacity [28]. Several studies on the *Campomanesia* genus have shown that a high phenolic content of the extracts and fractions is associated with significant antioxidant activity. The total phenolic content in the ethanol extract of *C. adamantium* leaves was 35.04%, presenting a DPPH free-radical scavenging activity with an EC_50_ of 13.00 μg/mL [29]. Studies on the ethanol extract of *C. pubescens* leaves showed high antioxidant activity using the DPPH assay [30]. Kataoka and Cardoso [31] assessed the antioxidant activity of *C. xanthocarpa* and *C. sessiliflora* leaves, showing a high content of total phenolics (138.15 μg/mL and 131.04 μg/mL, respectively) and a high percentage of DPPH free radical inhibition (54.52%, and 78.29%, respectively). Our results presented in this study demonstrate that the PEE has a high phenolic content associated with a high DPPH free-radical scavenging activity with EC_50_ 18.47 ± 3.74 μg/mL. These findings corroborate those described in the literature for the *Campomanesia* genus.

Pharmacological activities have been demonstrated for several species of *Campomanesia*, and they are related to their polyphenol content. The phytochemical profile of leaf hydroalcoholic extracts of *C. xanthocarpa* revealed the presence of flavonoids, saponins, and tannins, which were associated with the antiulcerogenic effect [17]. Other studies showed a reduction in proinflammatory markers, such as IL-6, IL-1, TNF-α, IFN-γ [32], antiplatelet, antithrombotic without cytotoxic effects and gastric lesions [33]. *C. adamantium* leaves exhibited anti-inflammatory and antinociceptive effects [34], antioxidant effects [26,29], and antimicrobial activity [27]. Previous studies of our research group identified several phenolic compounds in the phenolic-rich ethanol extract (PEE) of *C. lineatifolia* leaves, such as catechin, quercitrin, myricitrin, champanone D, licuroside, as well as terpenes, steroid, fatty acids and α-pyrone. This extract exhibited anti-inflammatory activity by inhibiting the NF-κB/TNF-α signalling pathway [20]. In addition, the ethanol extract and ethyl acetate fraction exhibited a gastroprotective activity in ethanol- and indomethacin-induced gastric lesion models [22]. Furthermore, the essential oil of *C. lineatifolia* leaves, which consists mainly of sesquiterpenes, presented an anti-*Helicobacter pylori* activity [18].

Considering the relationship between gastric ulcers, *H. pylori* infection, inflammation and the phenolic content of *C*. *lineatifolia*, in this study we set out to evaluate the anti-*H. pylori* activity of PEE, polar and non-polar fractions, MYR, MYR/QUER, and the tea. The results shown in Table 1 demonstrate that all tested samples could inhibit the growth in different strains of *H. pylori*.

PEE inhibited the growth of clarithromycin-sensitive type cultures (0.49 μg/mL), as well as clarithromycin-sensitive (125 μg/mL) and clarithromycin-resistant (250 μg/mL) clinical isolates. It is important to note that the PEE displays a concentration-dependent activity against *H. pylori* strains, when comparing clarithromycin-sensitive type cultures, clarithromycin-sensitive clinical isolates and clarithromycin-resistant clinical isolates, respectively. When considering plant extracts, MIC values < 500 μg/mL are deemed valuable and represent good antimicrobial activity [35]. These results represent a very interesting inhibitory activity against *H. pylori*, demonstrating that PEE has clinically relevant MIC values. This study investigated for the first time the anti-*H. pylori* of the phenolic-rich ethanol extract of *C. lineatifolia* and presents promising results for clinical use.

When the activities of isolated myricitrin from the extract were assessed, it was shown that this flavonol inhibited the growth of both clarithromycin-sensitive type cultures (62.5 and 250 μg/mL) and the clarithromycin-resistant clinical isolate (250 μg/mL), but not the clarithromycin-sensitive clinical isolate. Similarly to PEE, isolated myricitrin displays a concentration-dependent activity against *H. pylori* strains, when comparing clarithromycin-sensitive type cultures, and clarithromycin-resistant clinical isolates, respectively. In contrast, in the mixture of myricitrin and quercitrin, the main flavonoids in PEE, only the inhibition of the clarithromycin-sensitive clinical isolate was observed (31.25 μg/mL). Interestingly, these results complement each other and may suggest that the synergism of the phytocomplex is essential for a more robust inhibitory response to *H. pylori* growth. Although the isolated compounds displayed an inhibitory activity, concentrations above 10 μg/mL are not considered promising in the search for new antimicrobial agents [36].

It is important to note that for the polar and non-polar fractions obtained from the PEE, the *H. pylori* growth inhibition responses were similar in the clarithromycin-sensitive type cultures (MICs ranging from 0.49 to 250 μg/mL), the clarithromycin-sensitive (125 μg/mL, and 250 μg/mL, respectively) and -resistant clinical isolates (250 μg/mL, and 125 μg/mL, respectively). These results suggest that both polar and non-polar constituents in the *C. lineatifolia* extract are involved in the anti-*H. pylori* activity, corroborating the suggestion that the chemical matrix of *C. lineatifolia* is responsible for this activity.

As for the tea, the pharmaceutical form used by the population, the lowest concentration tested (1:100) was able to inhibit the growth of all the *H. pylori* strains, reaffirming the ethnopharmacological use of the *C. lineatifolia* leaves infusion in the treatment of gastric ulcers.

In Brazil, other species widely used in traditional medicine for the treatment or prevention of peptic ulcers have considerable anti-*H. pylori* activity. The antibacterial effect of aqueous garlic extract (*Allium sativum*) against *H. pylori* was investigated and exhibited a MIC value of 5 mg/mL. Also, it demonstrated a synergistic effect when combined with a proton pump-inhibitor (omeprazole) in a ratio of 250:1 [37]. A methanol extract of turmeric rhizome (*Curcuma longa*) and curcumin (isolated beta-diketone) both have a MIC within 6.25–50 μg/mL and are more effective against 5cagA+ *H. pylori* strains [38]. The anti-*H. pylori* activity demonstrated by the ginger-free phenolic and hydrolysed phenolic fractions of ginger (*Zingiber officinale*) exhibited MIC values of 38 ± 3.4 μg/mL, and 49 ± 4.1 μg/mL, respectively, against amoxicillin-sensitive clinical isolate strains [39]. MIC values of nonfermented and semifermented *C. sinensis* methanol: water extracts showed a bactericidal activity for *H. pylori* at 4 mg/mL and 5.5 mg/mL, respectively [40]. These results corroborate the anti-*H. pylori* activities of the *C. lineatifolia* samples tested in this study, even showing better activities when compared to the plants described above.

Several studies have shown anti-*H. pylori* activity associated with phenolic compounds. Voravuthikunchai, Limsuwan and Mitchell [41] associate the presence of phenolic compounds in *Punica granatum* (pomegranate) extracts with a change in the hydrophobicity of the *H. pylori* cell surface, which contributes to the inhibition of its growth. In another study, pomegranate peel extract exhibited remarkable activity against *H. pylori* in vitro with a MIC of 0.156 mg/mL, a pronounced urease inhibitory activity, and a synergic effect when combined with metronidazole against *H. pylori*, suggesting the species as a potential alternative or complementary therapy to treat *H. pylori* infection [42]. Takabayashi et al. [43] demonstrated that catechins present in green tea (*Camellia sinensis*) may have a bactericidal effect on *H. pylori* infection in an in vivo gerbil model.

Myricitrin and quercitrin have been reported to possess a wide spectrum of bioactivities, such as antioxidative, antimicrobial, immunomodulation and wound healing, which could be related to their anti-*H. pylori* activities. Since *H. pylori* stimulates antral mucosal reactive oxygen species (ROS) production [24], myricitrin and quercitrin, which have high antioxidant activity, may play an important role in reducing oxidative stress in *H. pylori* infections. Furthermore, myricitrin and quercitrin isolated from medicinal plants exhibit inhibitory activity against the *H. pylori* urease enzyme [44]. During the *H. pylori* infection, an intensive inflammation is developed in response to pathogen-associated molecular patterns (PAMPs) from *H. pylori*, and damage-associated molecular patterns (DAMPs) from damaged epithelial cells. PAMPs derived from *H. pylori* induce the NF-κB signalling pathway, and DAMPs from gastric epithelium induce inflammation, notably by interleukin-1 (IL-1), and tumor necrosis factor (TNF), pro-inflammatory cytokines [45]. A previous study conducted by our research group demonstrated that the PEE, isolated quercitrin and myricitrin from *C. lineatifolia* inhibited the TNF-α production in LPS-activated THP-1 cells. In addition, the PEE also was able to inhibit the NF-κB production [20]. When evaluating the beneficial effects on the swift healing of skin injuries, quercitrin and myricitrin were able to promote re-epithelization, fibroblast proliferation, collagen regeneration, and inflammatory factor attenuation, which suggests that these isolated flavonoids are promising molecules for wound treatment [46] and may contribute to regenerating the gastric mucosa infected by *H. pylori*. These results together suggest that the high phenolic content of the PEE, given especially by the major chemical markers quercitrin and myricitrin, contribute to a possible anti-inflammatory, anti-*H. pylori*, antioxidant and healing mechanism of the PEE from *C. lineatifolia*, which could justify its use in the treatment of gastric ulcers.

Importantly, the terpenoids identified in the polar fraction of *C. lineatifolia* [20] include hydroxy-acetylhydroshengmanol xylopyranoside, rubescensin M, and gingerglycolipid A, which have demonstrated, respectively, potential antimicrobial activity [47,48], traditional use in gastrointestinal disorders [49,50], and “stomachic” agent present in ginger, used in the treatment of gastric ulcers [51]. These results confirm the anti-*H. pylori* activity in association with the flavonoids catechin, quercitrin and myricitrin, and the minor terpenoids identified in *C. lineatifolia* PEE, polar fraction and the tea. As previously described, the PEE also showed anti-inflammatory activity by inhibiting the NF-κB/TNF-α signalling pathway [20], which may also contribute to reducing the inflammatory cascade and gastric damage, since *H. pylori* infection stimulates the activation of this signalling [52,53].

In addition to the flavonoids, nine minor compounds were previously identified in the non-polar fraction of the *C. lineatifolia* PEE, which we highlight here: a chalcone (licuroside), two β-triketones (champanone D and (iso)leptospermone), two terpenes (14α-hydroxy-15-acetoxysclareol and gaigrandine), two fatty acids (birsonic acid and heptanoic acid), a steroid (decumbesterone A) and an α-pyrone (cryptocaryol C) [20]. Licuroside is found in *Glycyrrhiza glabra* (liquorice) [54], and several studies have demonstrated the anti-*H. pylori* activity of its extracts [55,56,57,58]. Even when the liquorice is combined with the triple clarithromycin-based regimen, the result is an increase in *H. pylori* eradication [59]. Beta-triketones have demonstrated good antimicrobial activity [60,61]. The triterpene 14α-hydroxy-15-acetoxysclareol is described in *Eucalyptus sideroxylon* (Myrtaceae) and *Cimicifuga acerina* (Ranunculaceae), which are used in traditional medicine to treat inflammatory conditions, as well as being potential antimicrobial agents due to their constituents [47,48]. Gaigrandine was isolated and identified in *Helenium alternifolius* and *Gaillardia grandiflora*, and the relationship between sesquiterpene lactones and gastric cytoprotective activity was demonstrated [62,63]. Birsonic acid is described in *Byrsonima intermedia*, a plant with gastric and duodenal anti-ulcer activities, anti-*H. pylori* and antidiarrheal effects [64,65]. Heptanoic acid is the main constituent in *Acanthopanax brachypus*, traditionally used in Chinese medicine for various conditions, including gastric ulcers, and also has antibacterial activity [66,67]. The phytoecdysteroid decumbesterone A is described in *Ajuga decumbens*, and this species is traditionally used in medicine to treat inflammatory conditions, dysentery and gastrointestinal disorders [68,69]. Cryptocaryols are described in *Cryptocarya* species, and these α-pyrone derivatives show important anti-inflammatory activities [70].

These results together suggest that the combined chemical matrix described in this study for the extract of *C. lineatifolia* leaves may be responsible for the anti-*H. pylori* activity.

In 2017, the World Health Organization listed clarithromycin-resistant *H. pylori* in the high-priority category, requiring treatment attention. The main point of this problem is that resistance has been increasing sharply, and consequently, the success of therapeutic regimens that include clarithromycin for eradicating infection by this bacterium has been declining [71].

Recent protocols for treating clarithromycin-resistant *H. pylori* infection include first-line triple therapy with clarithromycin (i.e., a combination of 2nd-generation PPIs, amoxicillin and clarithromycin for 14 days) [72]. However, these regimens have several disadvantages, including serious adverse effects and high treatment costs, which make them difficult to implement in clinical practice and often lead to patients abandoning treatment. In addition, using multiple antimicrobial agents to treat *H. pylori* infection can increase the risk of future microbial resistance [73]. In this scenario, new regimens and approaches that allow minimal use of antimicrobials for a shorter treatment period are needed.

Herbal medicines effectively treat gastric ulcers and have fewer adverse effects and lower recurrence rates. Used in therapy alone or combined with conventional drugs, they can become an alternative for treating certain gastric ulcers, preventing their recurrence [6], and can dramatically increase the therapeutic effect of antibiotics [74]. Especially plants with antioxidant capability as the main mechanism are used as the herbal reservoir for treating ulcer disease [75]. Thus, the combination of herbal medicines with anti-*H. pylori* activity could improve the outcome for gastric ulcer patients. Considering the multiple targets of *C. lineatifolia* phenolic-rich ethanol extract, it is suggested that it has great potential in treating gastric ulcers associated with *H. pylori* infection.

## 4. Material and Methods

### 4.1. Chemicals and Reagents

The solvents hexane, ethyl acetate, methanol, butanol, acetic and formic acids were purchased from Trinity College Dublin HMF facilities (Dublin, Ireland). Ethanol 96 °C was acquired from Santa Cruz, Brazil. The standards, quercetin, quercitrin, myricitrin, pyrogallol, and rutin (all with purity ≥ 98%), hide powder, non-chromated, 2,2-diphenyl-1-picrylhydrazyl were purchased from Sigma-Aldrich, St. Louis, MO, USA. Sodium carbonate (Na_2_CO_3_), acetone, phosphomolybdic acid p.a., sodium tungstate p.a., acetic acid p.a., methanol UV/HPLC, aluminium chloride hexahydrate p.a., and anhydrous sodium sulphate were purchased from Dinâmica Química, Brazil. Ethyl acetate p.a. was acquired from Anadrol, Brazil; methenamine from Merck, Germany; ethanol p.a. from Exodo Científica, Brazil; and acetonitrile p.a. from LS Chemicals.

### 4.2. Plant Material

*Campomanesia lineatifolia* leaves were collected in February 2020 in Minas Gerais, Brazil (19°52′9.87″ S; 43°58′12.04″ W). The species was identified by Dr. Marcos Sobral from the Botany Department of Biological Sciences, Institute at Federal University of Minas Gerais (UFMG), Belo Horizonte. A voucher specimen (BHCB 150.606) was deposited at the UFMG Herbarium. Registration in the National System of Genetic Heritage and Associated Traditional Knowledge Management (SisGen) was carried out with the code A216C7C.

### 4.3. Phenolic-Rich Ethanol Extract

The phenolic-rich ethanol extract (PEE) was obtained as described by Neves et al. [19]. Briefly, *C. lineatifolia* leaves were dried at 40 °C/72 h with forced air circulation. The powdered dry leaves (5.0 g) were extracted using 100 mL of ethanol 96° by maceration under a continuous reflux extraction system (ball capacitor), T = 100 ± 5 °C for 3 cycles of 20 min. The ethanolic extract was centrifuged, filtered, and dried in a rotary evaporator (60 °C at 70 rpm), yielding 1.4087 g (28.17%) of dry extract.

### 4.4. Chemical Profile of Phenolic-Rich Extract by UHPCL

The extract solution obtained from *C. lineatifolia* PEE was prepared in 5 mg/mL concentration, as described next. A total of 5.0 mg of the PEE was weighed into plastic microtubes, and 1.0 mL of methanol analytical grade was added. Dissolution was performed under an ultrasonic bath for 20 min. Then, the solutions were centrifuged at 10,000× *g* for 10 min (Cientec Centrifuge, model CT-5000R). The supernatant was filtrated through a Millex (Millipore, Bedford, MA, USA) LCR (pore size, 0.45 μm) polytetrafluoroethylene membrane and transferred into 2.0 mL vials. Analyses were performed on a Waters UPLC Acquity System^®^ (Milford, MA, USA) equipped with a quaternary pump, autosampler, photodiode array detector, and Empower 2 Software Build 2154 SPs for data processing. An Acquity UPLC^®^ BEH C18 column (100 × 2.1 mm · 1.7 μm i.d.) and pre-column VanGuard ™ C18 (2.1 × 5 mm · 1.7 μm i.d.) was used at a temperature of 40 °C, flow rate of the mobile phase of 0.3 mL/min, and an injection volume of 2.0 μL. Ultraviolet (UV)-photodiode array detection was performed at λ 335 nm. UV spectra from λ 200 to 600 nm were recorded online for peak identification. The mobile phase consisted of two solvents: (A) 0.1% formic acid in ultrapure water (Millipore Direct-Q Water Purifier), and (B) 0.1% formic acid in acetonitrile. The following gradient program was performed: 0 min, 85% A–15% B; 9 min, 80% A–20% B; 9.1 min, 5% A–95% B; 11 min, 5% A–95% B; 12 min, 85% A–15% B; 15 min, 85% A–15% B.

### 4.5. Polar and Non-Polar Fractions

In order to obtain 4.0 g of the PEE, we carried out successive extractions of the powdered dry leaves using ethanol 96° by maceration under a continuous reflux extraction system, as described in Section 4.3 [19]. The PEE from leaves of *C. lineatifolia* (4.0 g) was fractionated using 50 mL of a hexane: ethyl acetate: methanol: water (HEMWat) solvent system in a ratio of 1:4:1:4, to obtain the upper and lower phases, or non-polar and polar fractions, respectively [20]. 

### 4.6. Aqueous Extract

Two litres of boiling ultrapure water were poured over 250 g of the powdered dry leaves, and the solution was subjected to an ultrasound bath for 20 min for solubilisation. Two consecutive filtrations were then carried out using cotton wool and filter paper, respectively, giving a final volume of 1 L of aqueous extract (tea).

### 4.7. Myricitrin and Myricitrin+Quercitrin

The polar fraction obtained from PEE *C. lineatifolia* leaves was subjected to high-speed countercurrent chromatography (HSCCC), followed by gel-filtration chromatography using a Sephadex LH-20 column. A complete purification was achieved allowing for the isolation of myricitrin (MYR), and a mixture of myricitrin plus quercitrin (MYR/QUER), identified by high-performance liquid chromatography coupled to electrospray ionisation and quadrupole time-of-flight mass spectrometry (HPLC-ESI-QTOF-MS), and nuclear magnetic resonance (NMR) [20].

### 4.8. Determination of Tannin Content

The method used to quantify tannins in the PEE was adapted from the Brazilian Pharmacopoeia 6th ed. [76], using the monograph of barbatimão (*Stryphnodendron adstringens*), as described by Barbosa [23]. The tannin content was estimated by calculating the difference between the total phenolic content and polyphenols not adsorbed by the hide-powder method.

The total phenolic content of the PEE was determined using the Folin–Denis assay, and the findings were expressed as grams of pyrogallol equivalents (PYE) per 100 g of samples (% *w*/*w*). In summary, 5 mL of diluted PEE solution (0.15 mg/mL) was mixed with 2 mL of Folin–Denis reagent and diluted to 50 mL in aqueous Na_2_CO_3_ solution (29% *w*/*v*). A standard curve was prepared using aqueous pyrogallol solutions ranging from 0.003125 mg/mL to 0.0375 mg/mL.

The hide-powder method was conducted by adding 0.2 g of hide-powder, non-chromated, to 20 mL of diluted PEE solution (0.15 mg/mL). After stirring for 60 min, the solution was filtered and a 5 mL aliquot was diluted to 25 mL in ultrapure water. Then, 5 mL of this solution was mixed with 2 mL of the Folin–Denis reagent and diluted to 50 mL in aqueous Na_2_CO_3_ solution (29% *w*/*v*).

The samples were kept in the dark at room temperature, and their absorbances were measured 30 min after adding Na_2_CO_3_ at λ 715 nm using an Infinite M200 Pro Spectrophotometer from Tecan, Switzerland. All samples were measured in triplicates.

### 4.9. Determination of Flavonoid Content

The method used to quantify flavonoids in the PEE was adapted from the Brazilian Pharmacopoeia 6th ed. [76], using the monograph for calendula (*Calendula officinalis*), as described by Barbosa [23]. The aluminium chloride colourimetric assay measured the total flavonoid content using the acid hydrolysis procedure. The results were expressed in grams of quercetin equivalents (QE) per 100 g of samples (% *w*/*w*).

The extractive solution was obtained by reflux extraction of 95 mg of the PEE in a 20 mL acidic acetone (10% HCl) solution in the presence of 1 mL methenamine (0.5%), for 30 min. The extract was cooled to room temperature (25 °C) and filtered using cotton wool, and the residue (cotton and plant material) was re-extracted with acetone twice more for 10 min. The filtered fractions were collected in a volumetric flask, and the volume was adjusted to 100.0 mL with the acetone. Fractionation was carried out by transferring a 20 mL aliquot of the extractive solution to a separatory funnel and adding 20 mL of ultrapure water. The solution was extracted once with 15 mL and thrice with 10 mL of ethyl acetate. The ethyl acetate phases were pooled and washed twice with 50 mL of water. The resulting ethyl acetate phase was filtered through filter paper, and 10.0 g of anhydrous sodium sulphate was added immediately before filtration to adsorb water residues. The filtrate was collected in a volumetric flask, and the volume was adjusted to 50.0 mL with the ethyl acetate. The resulting solution constituted the stock solution (SS). For analysis, 10 mL of SS was transferred to a volumetric flask, and 1.0 mL of aluminium chloride hexahydrate solution (2.0 g/100 mL of 5% *v*/*v* glacial acetic acid methanolic solution) was added. The volume was adjusted to 25.0 mL with 5% *v*/*v* glacial acetic acid methanolic solution.

A standard curve was prepared using 5% *v*/*v* glacial acetic acid methanolic quercetin solutions ranging from 0.001 mg/mL to 0.012 mg/mL. The samples were kept in the dark at room temperature, and their absorbances were measured 30 min after at λ 425 nm using an Infinite M200 Pro Spectrophotometer from Tecan, Switzerland. All samples were measured in triplicates.

### 4.10. Evaluation of Antioxidant Activity

The DPPH radical scavenging activity was carried out according to the method presented by Kurechi, Kikugawa and Kato [77], with adaptations. A total of 100 μL of various concentrations of extract (1.562 μg/mL to 50.0 μg/mL) were added to 40 μL of 300 μM DPPH radical solution in ethanol and incubated for 30 min in the dark at room temperature. The absorbance of the mixed solution was measured at λ 520 nm using a microplate reader (Infinite M200 Pro Spectrophotometer from Tecan, Switzerland). A rutin ethanol solution (100 μg/mL) was used as a positive control. DPPH radical scavenging activity was calculated using the equation below:

DPPH scavenging effect (%) = [(A_1_ − A_0_)/A_1_] × 100 (3)

A_1_ was the absorbance of the control (DPPH solution without sample) at λ 520 nm; A_0_ was the absorbance at λ 520 nm of the sample at different concentrations with DPPH.

The antioxidant activity was expressed as EC_50_ (μg/mL), the sample concentrations required to cause a 50% decrease in the absorbance at λ 520 nm. A lower EC_50_ value corresponded to a higher antioxidant activity.

### 4.11. Evaluation of Anti-Helicobacter pylori Activity

#### 4.11.1. Bacterial Strains and Culture Conditions

*H. pylori* strains were obtained from the American Type Culture Collection (ATCC 49503, clarithromycin-sensitive), National Collection of Type Cultures (NCTC11638, clarithromycin-sensitive), and from Tallaght University Hospital, Dublin 24, Ireland (SSR359, clarithromycin-sensitive clinical isolate, and SSR366, clarithromycin-resistant clinical isolate). All strains were maintained on Columbia blood agar (CBA) plates containing 5% sheep’s blood (VWR) at 37 °C under microaerobic conditions (CampyGen, Oxoid, Hampshire, UK). Bacteria were inoculated into Brain Heart Infusion (BHI) broth (Sigma) containing 10% foetal bovine serum (Gibco, Waltham, MA USA) and incubated under microaerobic conditions at 37 °C with shaking (120 rpm) for 24 h to prepare bacterial suspensions for broth microdilution assays [18].

#### 4.11.2. Broth Microdilution Assays

The determination of the minimum inhibitory concentration (MIC) of PEE, polar and non-polar fractions, MYR, MYR/QUER, and aqueous extract against *H. pylori* was evaluated using broth microdilution assays in 96-well plates, according to the protocol Standards for Antimicrobial Susceptibility Testing [78] with adaptations [79]. All the samples were diluted with BHI broth supplemented with 10% foetal bovine serum at 47 °C to prepare the concentrations at 0.49–1000 μL/mL (PEE, polar and non-polar fractions), 0.12–250 μL/mL (MYR, MYR/QUER), and 1:100, 1:50, 1:10 *v*/*v* (tea).

Then, 100 µL aliquots of overnight bacterial suspension (approximately 10^6^ CFU/mL; 1:20 0.5 McFarland Standard) [79] were added to each well of a 96-well plate. The cultures were incubated with 100 µL aliquots of the samples for 72 h at 37 °C under microaerobic conditions (CampyGen) with shaking (120 rpm). Absorbances at wavelength 600 nm were read using a Varioskan LUX Microplate Reader (Thermo Fisher Scientific). The MIC values were determined as the lowest concentration at which there was no bacterial growth. Clarithromycin was used as the positive control at 0.03–256 μg/mL (0.003–25.6% *v*/*v*).

### 4.12. Statistical Analysis

The experiments for anti-*H. pylori* activity were performed in triplicate, and the results were expressed as mean ± SEM. One-way ANOVA using GraphPad Prism 8.0 followed by the Bonferroni post-test were performed considering values of *p* ≤ 0.05 as statistically significant. MIC values were calculated considering the concentrations that did not differ statistically from the BHI broth control. Phenolic, tannin and flavonoid contents, and DPPH assays were expressed as mean ± SD. The coefficient correlation of standard curves was considered linear when R^2^ > 0.999.

## 5. Conclusions

As far as we know, this study shows the relationship between the high phenolic content, high antioxidant activity, and anti-*H. pylori* activity for *C. lineatifolia* species for the first time. The high anti-*H. pylori* activity of the phenolic-rich ethanol extract, the polar and non-polar fraction and the tea obtained from the leaves of *C. lineatifolia* show promising results for clinical use, and reinforces its popular use as a medicinal plant for the treatment of gastric ulcers. In addition, the activity observed against clinical isolates resistant to clarithromycin strengthens the potential use of the species as a coadjuvant in therapeutic regimes involving patients with resistant *H. pylori* infection. Evidence of the use of medicinal plants in therapy shows that they have fewer adverse effects and lower infection recurrence rates, in addition to reducing time and improving patient adherence to treatment. However, further studies are needed to propose the mechanisms by which the chemical compounds described here exert their anti-*H. pylori* activity.

## Figures and Tables

**Table 1:** Minimum inhibitory concentration (MIC) for *Campomanesia lineatifolia* samples against *Helicobacter pylori*. Concentrations tested: phenolic-rich ethanolic extract (PEE), polar and non-polar fractions 0.49–1000 μg/mL; myricitrin (MYR) and myricitrin+quercitrin (MYR/QUER) 0.12–250 μg/mL; and tea 1:100, 1:50, 1:10.

Samples	*Helicobacter pylori* Strains/MIC *			
	ATCC 49503 ^a^	NCTC 11638 ^a^	S SR359 ^b^	S SR366 ^c^
PEE	0.49 μg/mL	0.49 μg/mL	125 μg/mL	250 μg/mL
Polar fraction	500 μg/mL	0.49 μg/mL	125 μg/mL	250 μg/mL
Non-polar fraction	250 μg/mL	125 μg/mL	125 μg/mL	125 μg/mL
MYR	62.5 μg/mL	250 μg/mL	NI	250 μg/mL
MYR/QUER	NI	NI	31.25 μg/mL	NI
Tea	1:100	1:100	1:100	1:100
**Antimicrobial control**				
Clarithromycin	0.03 μg/mL	0.03 μg/mL	0.03 μg/mL	8 μg/mL

* One-way Anova, post-test Bonferroni, *p* < 0.05 (BHI control). ^a^ Clarithromycin-sensitive; ^b^ Clarithromycin-sensitive clinical isolate; ^c^ Clarithromycin-resistant clinical isolate; NI = non-inhibition.

## Data Availability

All data generated or analysed during this study are included in this article.
